# Supplementary material for: Variational quantum approximate support vector machine with inference transfer
Source: Sci Rep. 2023 Feb 25;13:3288. doi: 10.1038/s41598-023-29495-y (PMC9968349; doi:10.1038/s41598-023-29495-y)
Supplement: Supplementary file 1 — Supplementary Information. [file 41598_2023_29495_MOESM1_ESM.pdf]

Variational Quantum Approximate Support Vector Machine With Inference Transfer

Siheon Park[1], **Daniel K. Park**[2], **and June-Koo Kevin Rhee**[1,3,*]

[1]KAIST, School of Electrical Engineering, Daejeon, 34141, South Korea
[2]Yonsei University, Dept. of Applied Statistics & Dept. of Statistics and Data Science, Seoul, 03722, South Korea
[3]Qunova Computing, Inc., Deajeon, 34051, South Korea
[*]rhee.jk@kaist.edu

ABSTRACT

A kernel-based quantum classifier is the most practical and influential quantum machine learning technique for the hyper-linear classification of complex data. We propose a Variational Quantum Approximate Support Vector Machine (VQASVM) algorithm that demonstrates empirical sub-quadratic run-time complexity with quantum operations feasible even in NISQ computers. We experimented our algorithm with toy example dataset on cloud-based NISQ machines as a proof of concept. We also numerically investigated its performance on the standard Iris flower and MNIST datasets to confirm the practicality and scalability.

Supplementary Information

A Review of quantum support vector machine

Classification is a fundamental problem in machine learning. The goal of L-class classification is to infer the most likely class label of an unseen data point $\hat{\mathbf{x}} \in \mathbb{C}^N$ can be described as, given a labelled data set $\mathcal{S} = \{(\mathbf{x}_i, y_i)\}_{i=0}^{M-1} \subset \mathbb{C}^N \times \{0, 1, \ldots, L-1\}$ Although the data is real-valued in usual machine learning tasks, we allow complex-valued data without loss of generality. Support Vector Machine (SVM) is a supervised machine learning algorithm that classifies data to several classes by optimizing separating hyperplanes.[1,2] SVM is one of the most robust classifier for having global minimum guaranteed by convex optimization[3], and has been showing excellent performance not only in classification, but also in regression and other numerous fields. Also, it is known to be efficient for non-linear classification and regression for high-dimensional data with kernel trick. In this paper, we will focus on binary classification where only two class exists for the data (i.e. $L = 2$) since a multi-class classification can be achieved with multiple binary SVM by one-vs-all or one-vs-one scheme. Also, for notation simplicity, we assume $N = 2^n$, $M = 2^m$ $(n, m \in \mathbb{N}_0)$.

A.1 Hard-Margin SVM

For given dataset $\mathcal{S}$, $y \in \{-1, 1\}^N$, suppose the dataset is linearly separable, that is there exists a hyperplane $\langle \mathbf{w}, \mathbf{x} \rangle + b = 0$ such that

$$\begin{cases} \langle \mathbf{w}, \mathbf{x}_i \rangle + b \geq 1 & \forall y_i = 1 \\ \langle \mathbf{w}, \mathbf{x}_i \rangle + b \leq -1 & \forall y_i = -1 \end{cases} \tag{1}$$

The margin, distance between two parallel supporting hyperplanes, that we want to maximize is $2/\|\mathbf{w}\|$. Since $\arg\max 2/\|\mathbf{w}\| = \arg\min \|\mathbf{w}\|^2/2$, selecting optimal hyperplane $\langle \mathbf{w}^\star, \mathbf{x} \rangle + b^\star = 0$ and its parameters $(\mathbf{w}^\star, b^\star)$ is equivalent to solving a quadratic optimization problem (2).

$$p^\star = \min_{\mathbf{w}, b} \frac{1}{2} \|\mathbf{w}\|^2 : y_i(\langle \mathbf{w}, \mathbf{x}_i \rangle + b) \geq 1 \ \forall i \in \{0, \ldots, N-1\} \tag{2}$$

Instead of solving this primal problem, solving dual problem is much easier. The dual problem of (2) is

$$d^\star = \max_{\beta \geq 0} \left\{ \sum_{i=1}^{M-1} \beta_i - \frac{1}{2} \beta_i \beta_j y_i y_j \langle \mathbf{x}_i, \mathbf{x}_j \rangle \right\} : \sum_{i=0}^{M-1} \beta_i y_i = 0 \tag{3}$$

where the optimal solution of primal and dual problems satisfies Karush–Kuhn–Tucker conditions.

$$\mathbf{w}^\star = \sum_{i=1}^{M-1} \beta_i^\star y_i \mathbf{x}_i, \; \sum_{i=1}^{M-1} \beta_i^\star y_i = 0 \tag{4}$$

$$\beta_i^\star \left[y_i \left(\langle \mathbf{w}^\star, \mathbf{x}_i \rangle + b^\star \right) - 1 \right] = 0 \tag{5}$$

$$y_i \left(\langle \mathbf{w}^\star, \mathbf{x}_i \rangle + b^\star \right) \geq 1, \beta_i^\star \geq 0 \tag{6}$$

By investigating complementary slackness condition (5), it is clear that only the data on supporting hyperplanes can have non-zero $\beta_i^\star$ since $y_i \left(\langle \mathbf{w}^\star, \mathbf{x}_i \rangle + b^\star \right) = 1$ for those data. They are referred to as support vectors. Note that from this relationship one can find optimal bias that disappeared in dual problem.

$$b^\star = y_j - \sum_{i=1}^{M-1} \beta_i y_i \langle \mathbf{x}_i, \mathbf{x}_j \rangle \; \forall j : \alpha_j^\star > 0 \tag{7}$$

Since only support vectors are on supporting hyperplane, it can be considered as a set of linear combinations of support vectors. Thus, they are named as *support* vectors. The estimated label $\hat{y}$ of test data $\hat{\mathbf{x}}$ is determined by the relative location of test data with respect to the separating hyperplane $\{\mathbf{x} : \langle \mathbf{w}^\star, \mathbf{x} \rangle + b^\star = 0\}$.

$$\hat{y} = \operatorname{sgn} \left\{ \sum_{i=0}^{M-1} \langle \mathbf{x}_i, \hat{\mathbf{x}} \rangle + b^\star \right\} \tag{8}$$

From (8), we can treat Lagrange multiplier β as normalized weights of the labelled data.

A.2 Soft-Margin SVM

C-SVM introduce slack variables ξ_i's as violation between supporting hyperplane and outlier data in between two supporting hyperplanes. The goal is to minimize this violation while maximizing distance between supporting hyperplanes.

$$p^\star = \min_{\mathbf{w}, b, \xi} \frac{1}{2} \|\mathbf{w}\|^2 + \frac{C}{2} \sum_{i=1}^{M-1} \xi_i^2 : y_i \left(\langle \mathbf{w}, \mathbf{x}_i \rangle + b \right) \geq 1 - \xi_i \forall i \in \{0, \ldots, N-1\} \tag{9}$$

Here, hyperparameter C is user-defined value that controls over-fitting and under-fitting. We have adapted the formulation in Ref.[4] instead of original formulation in Ref.[2]. We can interpret the term $\frac{1}{2}\|\mathbf{w}\|^2$ as regularizing term, and $1/C$ as its hyperparameter. With the same logic in case of hard-margin SVM, solving dual problem is much easier. The dual problem of (9) is

$$d^\star = \max_{\beta \geq 0} \sum_{i=1}^{M-1} \beta_i - \frac{1}{2} \sum_{i,j=0}^{M-1} \beta_i \beta_j y_i y_j \langle \mathbf{x}_i, \mathbf{x}_j \rangle - \frac{1}{2C} \sum_{i=0}^{M-1} \beta_i^2 : \sum_{i=0}^{M-1} \beta_i y_i = 0 \tag{10}$$

where the optimal solution of primal and dual problems satisfies Karush–Kuhn–Tucker conditions.

$$\mathbf{w}^\star = \sum_{i=1}^{M-1} \beta_i^\star y_i \mathbf{x}_i, \; \sum_{i=1}^{M-1} \beta_i^\star y_i = 0, \xi_i^\star = \frac{1}{C} \beta_i^\star \tag{11}$$

$$\alpha_i^\star \left[y_i \left(\langle \mathbf{w}^\star, \mathbf{x}_i \rangle + b^\star \right) - 1 + \xi_i^\star \right] = 0 \tag{12}$$

$$y_i \left(\langle \mathbf{w}^\star, \mathbf{x}_i \rangle + b^\star \right) \geq 1 - \xi_i^\star, \; \alpha_i^\star \geq 0 \tag{13}$$

By investigating complementary slackness condition in (12), it is clear that only the data on and in between supporting hyperplanes can have non-zero $\beta_i^\star$ since $y_i \left(\langle \mathbf{w}^\star, \mathbf{x}_i \rangle + b^\star \right) = 1 - \xi_i$ for those data. Therefore, with the same logic as before, optimal bias is obtained manually.

$$b^\star = y_q (1 - C^{-1} \beta_q^\star) - \sum_{i=0}^{M-1} \beta_i^\star y_i k(\mathbf{x}_q, \mathbf{x}_i) \tag{14}$$

However, there may be no examples exactly lying on the supporting hyperplane. In this case, we can approximate $b^\star$ as median value of absolute difference $|y_i - \langle \mathbf{w}^\star \mathbf{x}_i \rangle|$ among all training data.[5] Estimating test label would be the same as that of the Hard-margin SVM (8).

A.3 Kernel Trick

Even with the soft margin assumption, dataset may not be linearly separated efficiently. In this case, we can define feature map $\phi : \mathcal{X} \mapsto \mathcal{H}$ to map data to high dimensional Hilbert space $\mathcal{H}$. With the sophisticated feature map, we expect mapped dataset $\mathbf{x} \to \phi(\mathbf{x})$ is linearly separable in $\mathcal{H}$.

$$d^\star = \max_{\beta \geq 0} \sum_{i=1}^{M-1} \beta_i - \frac{1}{2} \sum_{i,j=0}^{M-1} \beta_i \beta_j y_i y_j \langle \phi(\mathbf{x}_i), \phi(\mathbf{x}_j) \rangle - \frac{1}{2C} \sum_{i=0}^{M-1} \beta_i^2 : \sum_{i=0}^{M-1} \beta_i y_i = 0 \tag{15}$$

$$\hat{y} = \operatorname{sgn} \left\{ \sum_{i=0}^{M-1} \langle \phi(\mathbf{x}_i), \phi(\hat{\mathbf{x}}) \rangle + b^\star \right\} \tag{16}$$

However, defining feature map may not be practical nor possible. For example, the dimension of $\mathcal{H}$ should be infinite in order to linearly separate any mapped dataset. In addition, the *closeness* between two mapped data is not clear. Note that both Equation (15) and (16) require calculation of inner product between ϕ instead of ϕ itself. Thus, rather than constructing ill-defined vector map, we define kernel function $k : \mathcal{X} \times \mathcal{X} \mapsto \mathbb{C}$.

$$k(\mathbf{x}, \mathbf{y}) = \langle \phi(\mathbf{x}), \phi(\mathbf{y}) \rangle_{\mathcal{H}} \tag{17}$$

We can consider the kernel as the measure of *similarity*, for it is defined as inner product between mapped vectors. It has been proven that there exists unique feature map ϕ for positive semi-definite kernel $k(\cdot,\cdot) = \langle \phi(\cdot), \phi(\cdot) \rangle_{\mathcal{H}}$. Consider the formulations (10) and (8). They imply that the inner product between examples only matter for given dataset. Therefore, if we define some features $\phi(\mathbf{x}_i)$ to represent $\mathbf{x}_i$, then the dual SVM formulation (10) and classifying equation (8) are almost the same except for inner product part.

Since $\phi(\cdot)$ can be highly non-linear mapping, we can construct non-linear classifier on given dataset using SVM. Note that SVM only solves linear classification problem originally. Users can now have more degree of freedom from the arbitrary selection on the feature mapping to classify examples. However, $\phi(\cdot)$ may map original data to very high-dimensional Hilbert space so that calculating not only features themselves but also inner product of features explicitly may cost severe computation resources. It has been proven that there exist a unique feature map for any positive semi-definite (PSD) kernel. Therefore users can define a kernel to solve dual SVM problem. This is known as kernel trick[2,5,6].

$$d^\star = \max_{\beta \geq 0} \sum_{i=1}^{M-1} \beta_i - \frac{1}{2} \sum_{i,j=0}^{M-1} \beta_i \beta_j y_i y_j k(\mathbf{x}_i, \mathbf{x}_j) - \frac{1}{2C} \sum_{i=0}^{M-1} \beta_i^2 : \sum_{i=0}^{M-1} \beta_i y_i = 0 \tag{18}$$

$$\hat{y} = \operatorname{sgn} \left\{ \sum_{i=0}^{M-1} k(\mathbf{x}_i, \hat{\mathbf{x}}) + b^\star \right\} \tag{19}$$

There are several popular choice for kernel; polynomial kernel and Gaussian radial basis function kernel, for examples,

$$k(\mathbf{x}, \mathbf{y}) = (\langle \mathbf{x}, \mathbf{y} \rangle + c)^d, \tag{20}$$

$$k(\mathbf{x}, \mathbf{y}) = e^{-\frac{\gamma}{2} \|\mathbf{x}-\mathbf{y}\|^2}. \tag{21}$$

Polynomial kernel is defined as (20) with kernel hyperparameter bias c, and order d. Gaussian radial basis function kernel(RBF kernel) is defined as (21) with kernel hyperparameter inverse of standard deviation γ.

B Proof of Eqs. (15) and (16)

The proof of Eqs. (15) is as follows:

$$
\begin{aligned}
|\psi\rangle &= \sum_{i=0}^{M-1} \sqrt{\alpha_i}\,|0,i,\phi(\mathbf{x}_i),y_i,\phi(\hat{\mathbf{x}})\rangle \\
&\Longrightarrow \frac{1}{\sqrt{2}}\sum_{i=0}^{M-1} \sqrt{\alpha_i}\,|0,i,\phi(\mathbf{x}_i),y_i,\phi(\hat{\mathbf{x}})\rangle + \frac{1}{\sqrt{2}}\sum_{i=0}^{M-1} \sqrt{\alpha_i}\,|1,i,\phi(\mathbf{x}_i),y_i,\phi(\hat{\mathbf{x}})\rangle \\
&\Longrightarrow \frac{1}{\sqrt{2}}\sum_{i=0}^{M-1} \sqrt{\alpha_i}\,|0,i,\phi(\mathbf{x}_i),y_i,\phi(\hat{\mathbf{x}})\rangle + \frac{1}{\sqrt{2}}\sum_{i=0}^{M-1} \sqrt{\alpha_i}\,|1,i,\phi(\hat{\mathbf{x}}),y_i,\phi(\mathbf{x}_i)\rangle \\
&\Longrightarrow \frac{1}{2}\sum_{i=0}^{M-1} \sqrt{\alpha_i}\left(|0,i,\phi(\mathbf{x}_i),y_i,\phi(\hat{\mathbf{x}})\rangle + |0,i,\phi(\hat{\mathbf{x}}),y_i,\phi(\mathbf{x}_i)\rangle\right) \\
&\quad + \frac{1}{2}\sum_{i=0}^{M-1} \sqrt{\alpha_i}\left(|1,i,\phi(\mathbf{x}_i),y_i,\phi(\hat{\mathbf{x}})\rangle - |1,i,\phi(\hat{\mathbf{x}}),y_i,\phi(\mathbf{x}_i)\rangle\right) \\
&\quad \xrightarrow{\langle Z_a Z_y\rangle} \sum_{i=0}^{M-1} \alpha_i y_i |\langle\phi(\mathbf{x}_i)|\phi(\hat{\mathbf{x}})\rangle|^2,\ \xrightarrow{\langle I_a Z_y\rangle} \sum_{i=0}^{M-1} \alpha_i y_i
\end{aligned}
\tag{22}
$$

The proof of Eqs. (16) is as follows:

$$
\begin{aligned}
|\psi\rangle &= \sum_{i,j=0}^{M-1} \sqrt{\alpha_i}\sqrt{\alpha_j}\,\left|0,i,\phi(\mathbf{x}_i),y_i,j,\phi(\mathbf{x}_j),y_j\right\rangle \\
&\Longrightarrow \frac{1}{\sqrt{2}}\sum_{i,j=0}^{M-1} \sqrt{\alpha_i}\sqrt{\alpha_j}\,\left|0,i,\phi(\mathbf{x}_i),y_i,j,\phi(\mathbf{x}_j),y_j\right\rangle + \frac{1}{\sqrt{2}}\sum_{i,j=0}^{M-1} \sqrt{\alpha_i}\sqrt{\alpha_j}\,\left|1,i,\phi(\mathbf{x}_i),y_i,j,\phi(\mathbf{x}_j),y_j\right\rangle \\
&\Longrightarrow \frac{1}{\sqrt{2}}\sum_{i,j=0}^{M-1} \sqrt{\alpha_i}\sqrt{\alpha_j}\,\left|0,i,\phi(\mathbf{x}_i),y_i,j,\phi(\mathbf{x}_j),y_j\right\rangle + \frac{1}{\sqrt{2}}\sum_{i,j=0}^{M-1} \sqrt{\alpha_i}\sqrt{\alpha_j}\,\left|1,i,\phi(\mathbf{x}_j),y_i,j,\phi(\mathbf{x}_i),y_j\right\rangle \\
&\Longrightarrow \frac{1}{2}\sum_{i,j=0}^{M-1} \sqrt{\alpha_i}\sqrt{\alpha_j}\left(\left|0,i,\phi(\mathbf{x}_i),y_i,j,\phi(\mathbf{x}_j),y_j\right\rangle + \left|0,i,\phi(\mathbf{x}_j),y_i,j,\phi(\mathbf{x}_i),y_j\right\rangle\right) \\
&\quad + \frac{1}{2}\sum_{i,j=0}^{M-1} \sqrt{\alpha_i}\sqrt{\alpha_j}\left(\left|0,i,\phi(\mathbf{x}_i),y_i,j,\phi(\mathbf{x}_j),y_j\right\rangle - \left|0,i,\phi(\mathbf{x}_j),y_i,j,\phi(\mathbf{x}_i),y_j\right\rangle\right) \\
&\quad \xrightarrow{\langle Z_a Z_{y_0} Z_{y_1}\rangle} \sum_{i,j=0}^{M-1} \alpha_i\alpha_j y_i y_j \left|\left\langle\phi(\mathbf{x}_i)\middle|\phi(\mathbf{x}_j)\right\rangle\right|^2,\ \xrightarrow{\langle I_a Z_{y_0} Z_{y_1}\rangle} \sum_{i,j=0}^{M-1} \alpha_i\alpha_j y_i y_j = \left(\sum_{i=0}^{M-1} \alpha_i y_i\right)^2
\end{aligned}
\tag{23}
$$

For more detail, see[7,8].

C Supplementary Figures

In this section, we provide supplementary figures mentioned in the main text.

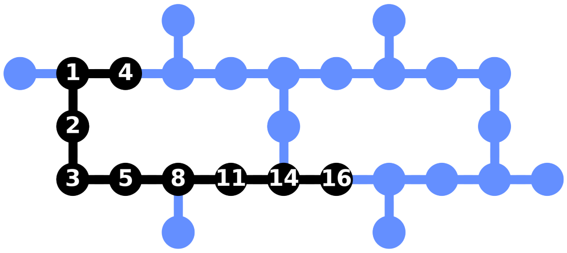

Figure S1. *ibm_montreal* qubits configurations. We used qubits colored in black for QASVM with toy data set

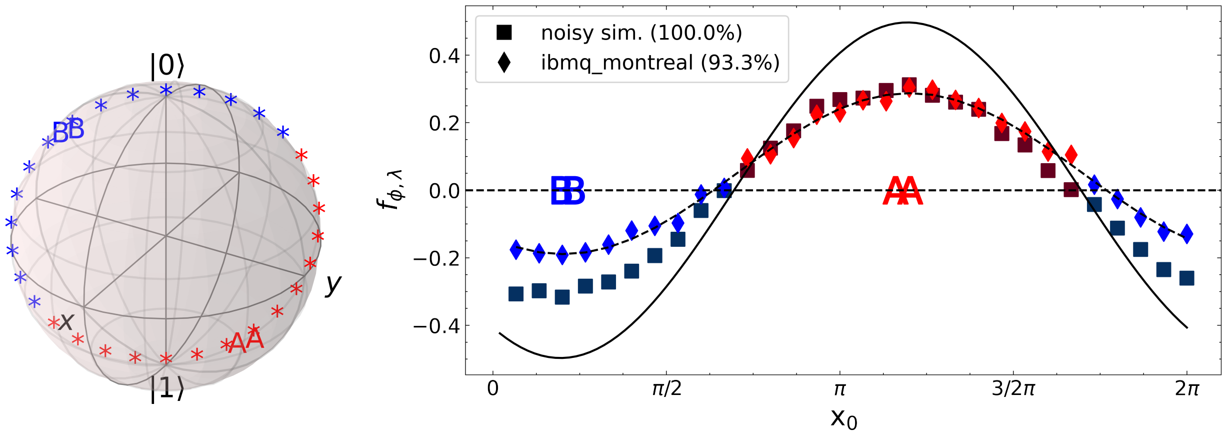

a Balanced toy dataset

b Classification result

Figure S2. Experiments on *ibmq_montreal* cloud NISQ processor with different example dataset. Here, the training data set is balanced.

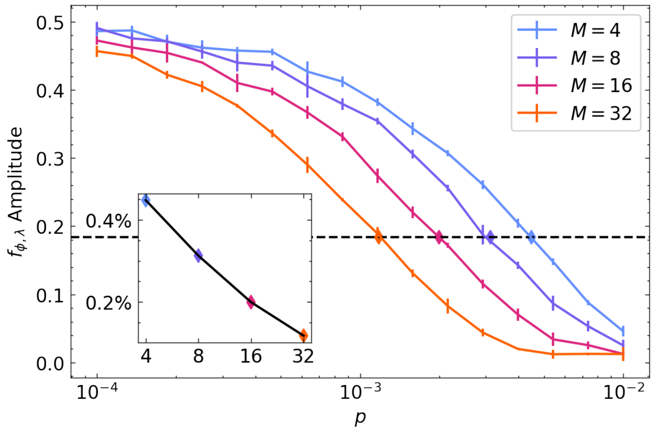

Figure S3. Noise robustness simulation result on depolarizing error. $f_{\phi,\lambda}$ amplitude represents amplitude of sine-fitted function of measured decision values. p is depolarizing error rate of single-qubit gate. We set the error rate of two-qubit gates 10 times higher than that of single-qubit gates.

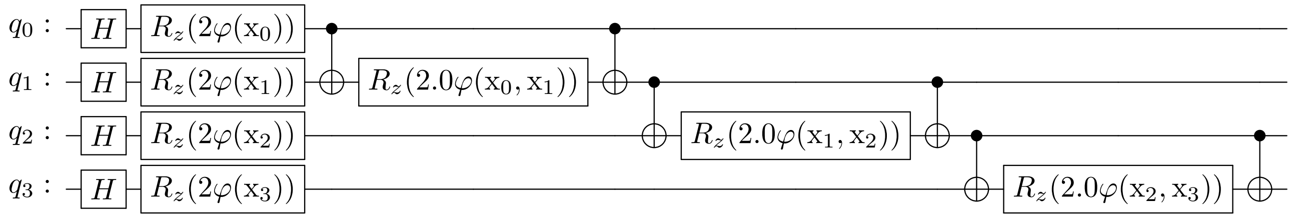

a *ZZFeatureMap* with linear entanglement

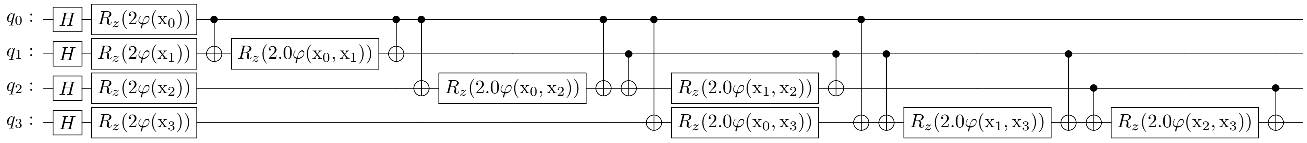

b *ZZFeatureMap* with all-to-all entanglement

Figure S4. *ZZfeatureMap* quantum feature map from Ref.[9]

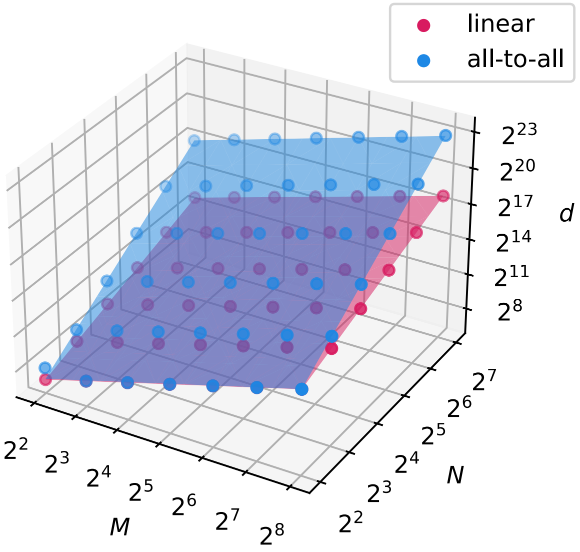

a Loss circuit depth

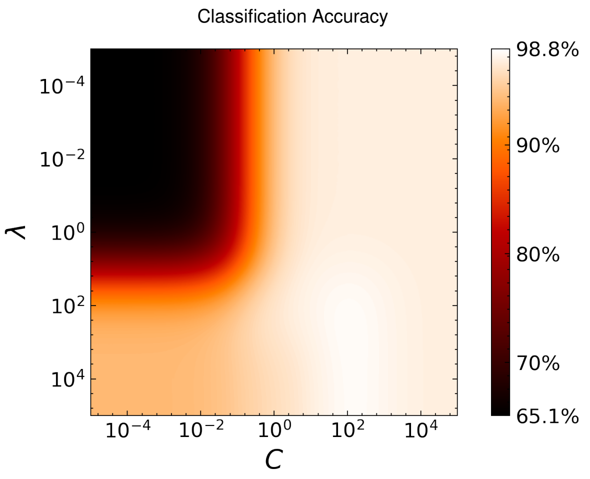

b Hyperparameter selection

Figure S5. Numerical order analysis. **a**, depth of quantum circuit d. The closed circles indicate simulated samples, where transparent planes are the linear fitting. In the legend, 'linear' indicates the use of the feature map in Fig. S4a, and 'all-to-all' indicates the use of the feature map in Fig. S4b. The results show linear dependence on M. The circuits are decomposed with a universal gate set $\{R_x, R_y, R_z, CNOT\}$. **b**, classification accuracy of iris data set with a $M = 64$ training data set displayed as a function of λ and C.

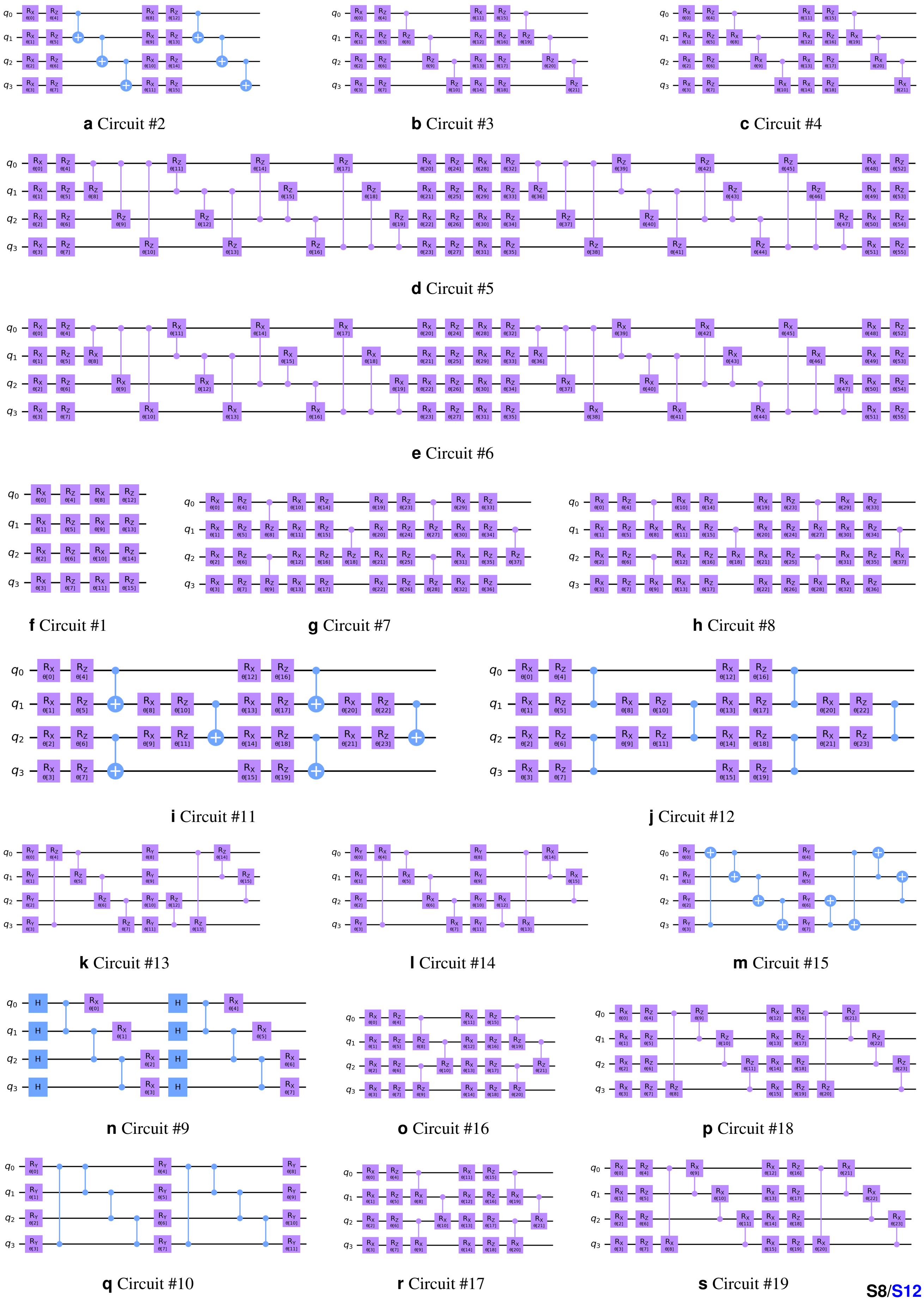

Figure S6. 19 sample PQC architecture provided in Ref. 10. Each circuit has 4 qubits and 2 layers.

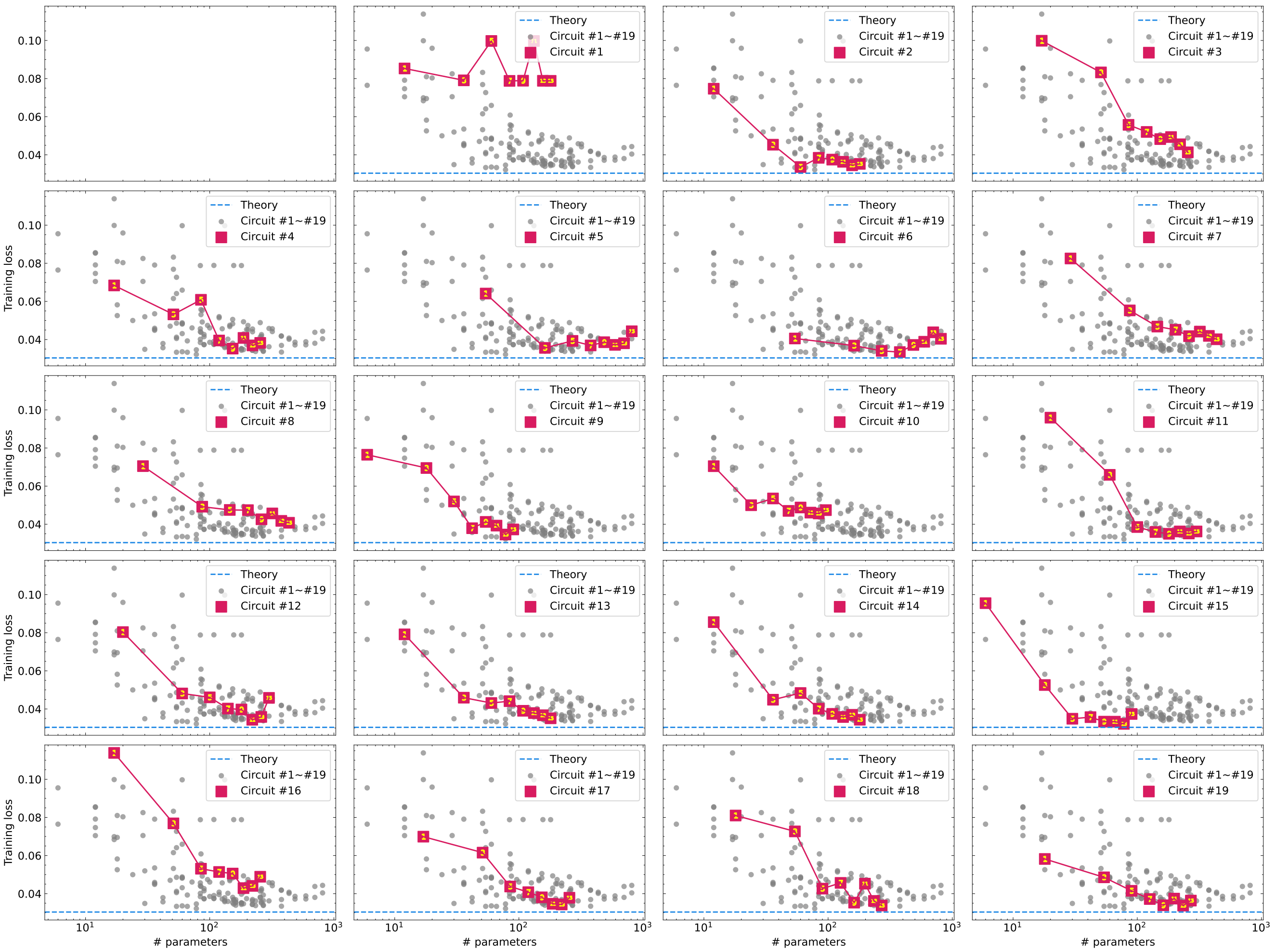

Figure S7. Training results on iris data set ($M = 64$)

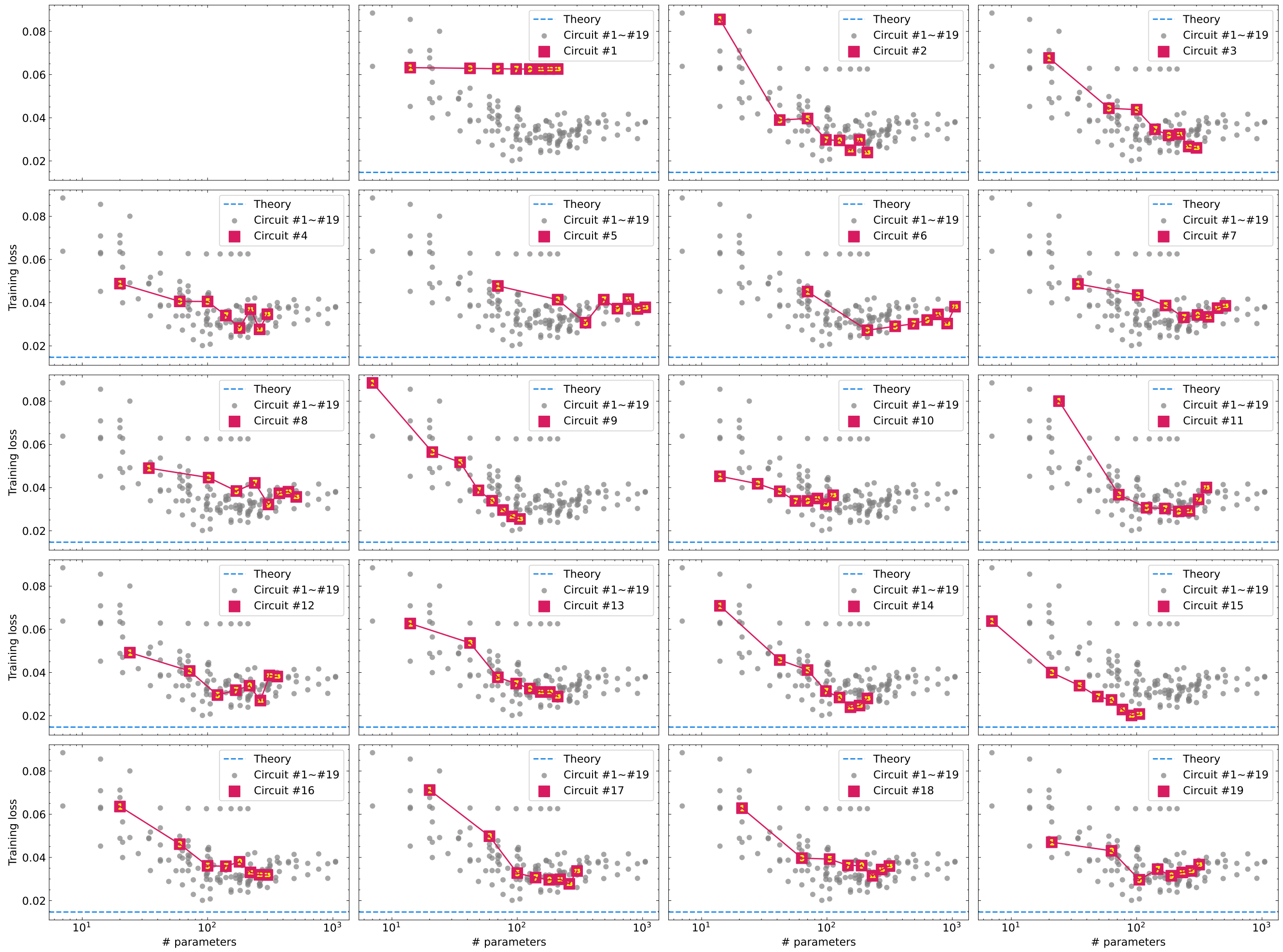

Figure S8. Training results on iris data set ($M = 128$)

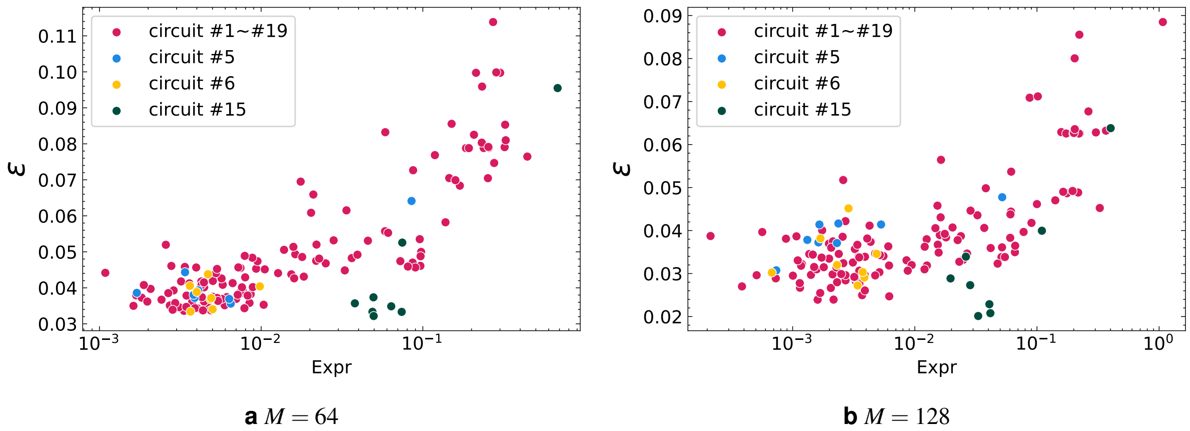

Figure S9. Decision value error on iris data set. Expr refers to expressibility of PQCs.[10] Circuit #5 and #6 has $L \times \log(M)^2$ parameters.

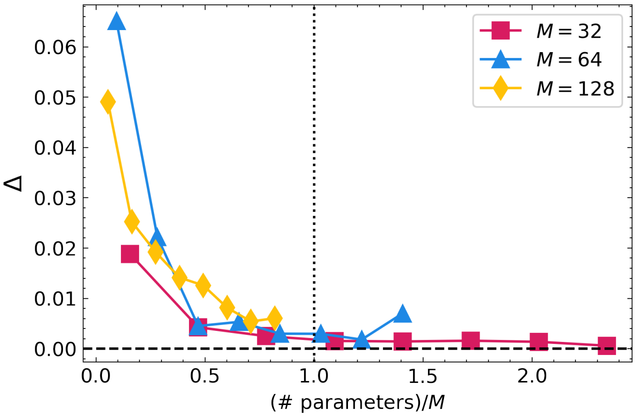

a Loss on training data

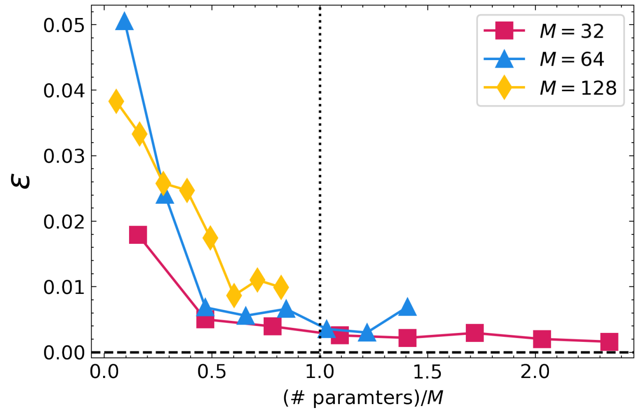

b Accuracy on test data

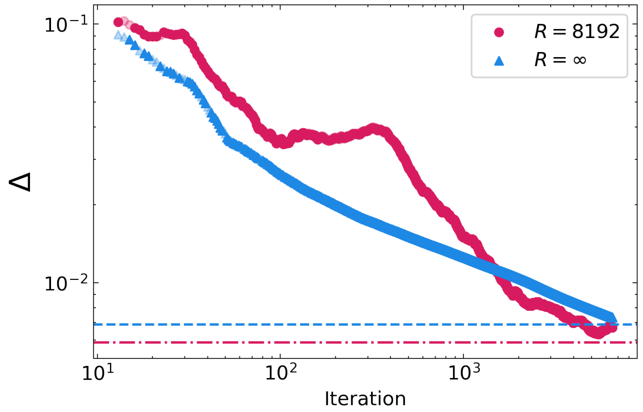

c Training loss convergence

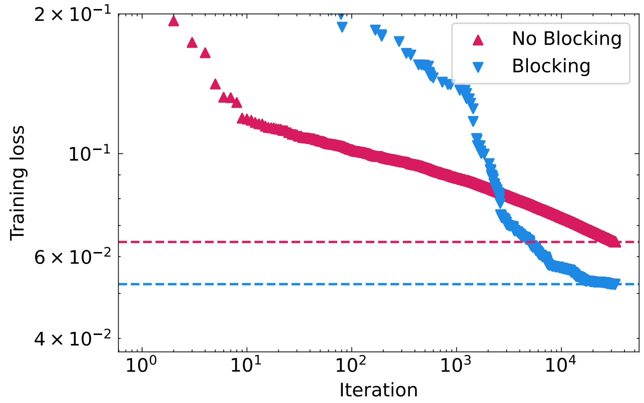

d Blocking technique for SPSA

Figure S10. Numerical Analysis on iris data set ($\lambda = C = 10^4$). In **a** and **b**, after 2^{10} iterations, the residual loss of training Δ and average decision value error $\mathcal{E}$ are plotted as a function of normalized number of parameters with respect to M, respectively. In **c**, coarse-grained Δ averaged over $10^{-0.1}t$ to $10^{0.1}t$ at iteration t is shown in a log scale plot. In **d**, Training loss converges faster when blocking technique for SPSA is used.

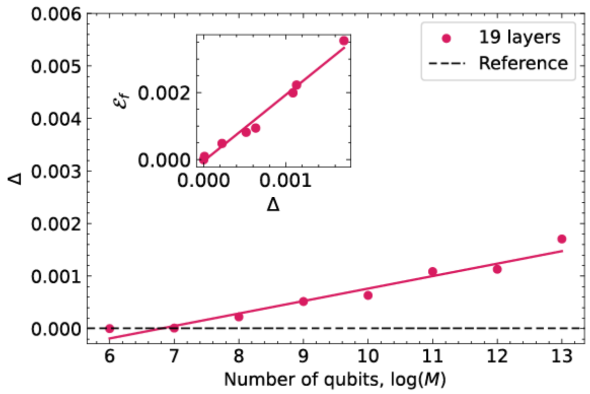

a Trainability

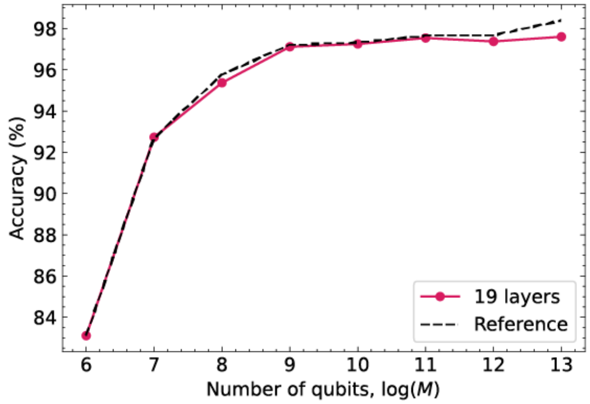

b Classification Accuracy

Figure S11. Numerical Analysis on the MNIST dataset ($\lambda = C = 10^4$). In the legend, '19 layers' refers to the results of VQASVM with the PQC having 19 layers, whereas 'Reference' denotes the results obtained by convex optimization. **(a)** $\mathcal{E}_f$ is the error on decision function; $\mathcal{E}_f = \sum_{\hat{\mathbf{x}} \in \mathcal{T}} |f_{\lambda,\phi}(\hat{\mathbf{x}}; \theta^\star, \mathcal{S}) - h(\hat{\mathbf{x}}; \mathcal{S})| / |\mathcal{T}|$ where $\mathcal{T}$ is the set of test data and h is the theoretical decision function obtained by convex optimization.

References

1. Boser, B. E., Guyon, I. M. & Vapnik, V. N. A training algorithm for optimal margin classifiers. In *Proceedings of the fifth annual workshop on Computational learning theory*, 144–152 (1992).
2. Cortes, C. & Vapnik, V. Support-vector networks. *Mach. Learn.* **20**, 273–297, DOI: 10.1007/BF00994018 (1995).
3. Steinwart, I. & Christmann, A. *Support vector machines* (Springer Science & Business Media, 2008).
4. Liu, Y., Arunachalam, S. & Temme, K. A rigorous and robust quantum speed-up in supervised machine learning. *Nat. Phys.* **17**, 1013–1017, DOI: 10.1038/s41567-021-01287-z (2021).
5. Deisenroth, M. P., Faisal, A. A. & Ong, C. S. *Mathematics for Machine Learning* (Cambridge University Press, 2020).
6. Boyd, S. P. & Vandenberghe, L. *Convex optimization* (Cambridge university press, 2004).
7. Blank, C., Park, D. K., Rhee, J.-K. K. & Petruccione, F. Quantum classifier with tailored quantum kernel. *npj Quantum Inf.* **6**, 41, DOI: 10.1038/s41534-020-0272-6 (2020).
8. Park, D. K., Blank, C. & Petruccione, F. The theory of the quantum kernel-based binary classifier. *Phys. Lett. A* **384**, 126422, DOI: 10.1016/j.physleta.2020.126422 (2020).
9. Havlíček, V. *et al.* Supervised learning with quantum-enhanced feature spaces. *Nature* **567**, 209–212, DOI: 10.1038/s41586-019-0980-2 (2019).
10. Sim, S., Johnson, P. D. & Aspuru-Guzik, A. Expressibility and Entangling Capability of Parameterized Quantum Circuits for Hybrid Quantum-Classical Algorithms. *Adv. Quantum Technol.* **2**, 1900070, DOI: 10.1002/qute.201900070 (2019).
